# Prior contact with permethrin decreases its irritancy at the following exposure among a pyrethroid-resistant malaria vector *Anopheles gambiae*

**DOI:** 10.1038/s41598-019-44633-1

**Published:** 2019-06-03

**Authors:** Margaux Mulatier, Cédric Pennetier, Angélique Porciani, Fabrice Chandre, Laurent Dormont, Anna Cohuet

**Affiliations:** 1 MIVEGEC, IRD, CNRS, Univ. Montpellier, Montpellier, France; 2 CEFE, Univ Paul Valéry Montpellier 3, CNRS, Univ Montpellier, EPHE, IRD, Montpellier, France; 3 Institut Pierre Richet, Bouaké, Côte d’Ivoire

**Keywords:** Behavioural ecology, Malaria, Public health

## Abstract

Insecticide-treated nets (ITNs) remain major components for vector control despite the spread of resistance mechanisms among mosquito populations. Multiple exposures to pyrethroids may induce physiological and behavioral changes in mosquitoes, possibly reducing efficacy of control tools. Despite epidemiological relevance, the effects of multiple exposures to pyrethroids on their efficacy against pyrethroid-resistant mosquitoes has received little interest. In the present study, we assessed the effects of a blood-meal successfully obtained upon a permethrin-treated net on the success at taking a second blood-meal in presence of permethrin in *Anopheles gambiae*, carrying pyrethroid resistance alleles. We also measured the impact of exposure to permethrin on life-history traits to address the delayed efficacy of ITNs. Our results showed that females that successfully blood-fed upon a permethrin-treated net were no longer inhibited by permethrin at the following exposure. Blood-meal inhibition due to permethrin was not affected by female size nor by exposure of mothers when testing the offspring, allowing to discard the effect of genetic or physiological selection. Besides, in our assays, exposure to permethrin did not affect mosquito fecundity, fertility nor survival. These results give insights to understand the long-term efficacy of ITNs, and allow to reevaluate the criteria used when choosing compounds for fighting malaria mosquitoes.

## Introduction

Mosquito-borne diseases are responsible for 700,000 deaths every year, with communities with poor-living conditions being the most affected^1^. Vector control plays a key role in the fight against these diseases, and mostly relies on the use of insecticides. Particularly, insecticide-treated nets (ITNs) and indoor residual spraying (IRS) are the primary and almost the single tools available to protect people against mosquito bites^2^. The extensive use of insecticides both in vector control and agriculture has triggered considerable selective pressure on mosquito populations^3–6^, inducing the spread of multiple resistance mechanisms among them^7^. For instance, *kdr* mutations are now distributed worldwide and confer resistance to pyrethroids^2,8–10^, the main class of insecticides used for vector control and the single allowed on nets impregnation up to now^2,11^. The propagation of resistance mechanisms not only threatens the efficacy of the current control tools^12–14^, but also indirectly favors the resurgence and explosive outbreaks of mosquito borne diseases, imposing a serious threat to public health programs^15–17^.

To date, alternatives to replace insecticides in the fight against vector-borne diseases remain limited. In consequence, insecticides still prevail as the main tool for protecting people against vector-borne diseases. This is particularly true in the case of pathogens transmitted by nocturnal mosquitoes such as malaria parasites, against which pyrethroid-impregnated nets are expected to provide physical and chemical barrier against mosquitoes^18^. Pyrethroid-treated nets are also thought to remain efficient to some extent against pyrethroid-resistant mosquitoes, due to their intrinsic characteristics of contact irritancy^12,13,19^, the interactions with additional stresses such as parasite infection^20,21^, and the possible delayed mortality they induce^22^. Such remaining efficacy of ITNs against mosquitoes considered as pyrethroid-resistant may explain the observed paradox between the dramatic spread of resistance alleles among vector populations and the conserved efficacy of ITNs at epidemiological level^23^. In the context of intense use of insecticides for vector control, the residual efficacy of pyrethroids (*i.e*. the efficacy beyond exposure) among vector populations that carry resistance mechanisms deserves great attention. However, very little has been dedicated to the effects of multiple exposures to pyrethroids on mosquito physiology and their consequences on long-term ITNs efficacy. Of special interest, as mosquitoes get infected during blood-feeding, infectious mosquitoes are expected to be more prone to have experienced prior contact with pyrethroid-treated nets than young and non-infectious individuals in areas of high ITNs cover.

Multiple exposures to pyrethroids among resistant populations could affect several physiological mosquito mechanisms, such as sensory detection of the chemical, nervous integration of the stimuli, and/or chemical detoxification^24–27^. For instance, cumulative exposures to pyrethroids may negatively affect mosquito physiology, increasing their efficacy over time. This has been observed in susceptible strains of *Anopheles* mosquitoes, where a prior sub-lethal exposure to pyrethroids resulted in an increased susceptibility after 24 hours^28^. Also, ITNs efficacy could remain constant along mosquito lifespan, as it was documented in susceptible^29^ and insecticide-resistant mosquitoes^22^. Besides, pyrethroids, and particularly permethrin, are known to induce irritant effects on mosquitoes, and to act on mosquito behavior^30^. Multiple exposures to pyrethroids could then affect mosquito behavior over time and its irritability when contacting pyrethroids. These changes could reduce pyrethroids residual efficacy along the lifespan of resistant mosquitoes. Finally, multiple exposures to pyrethroids could trigger habituation and complex learning mechanisms, reducing their efficacy over time, as it was previously observed with repellents^31,32^.

Multiple exposures to insecticides may also affect mosquito life-history traits as it has been reported in insecticide-susceptible insects^33–35^. Interestingly, to our knowledge, this has never been addressed in insecticide-resistant mosquitoes. Also, insecticide efficacy is rarely assessed considering blood-feeding inhibition, which is nonetheless a biologically relevant parameter directly related to parasite transmission. To this regard, looking beyond the first-contact efficacy and considering insecticide resistance is of crucial interest for understanding the implications of ITNs use for controlling pathogen transmission, as well as the interactions between ITNs and pyrethroid resistance.

In the present study, we measured the effect of a successful blood-meal obtained upon a permethrin-treated net on the success at blood-feeding despite the presence of permethrin at the subsequent exposure, 3 or 4 days later, in the malaria mosquito *An. gambiae* carrying pyrethroid resistance alleles. We also measured the effect of permethrin on mosquito fitness after the first and second bloodmeal, to assess the impact of permethrin exposure on mosquitoes’ life history traits.

## Results

For the first exposure, 1521 females were provided a blood-meal in presence of permethrin, across 7 replicates. To do this, they were given access to a blood-meal upon a net treated with permethrin at a dose of 500 mg/m², so they must contact the treated net to blood-feed. 692 females were exposed to net treated with ethanol (*i.e*. control) and displayed blood-feeding rates of about 73% (95% CI [48.42–96.93]). The net impregnation with permethrin significantly affected blood-feeding at the first exposure, with a mean inhibition of 37% (95% CI [6.89–66.34]) compared to the control group (*X*² = 246.27, Df = 1, P < 2.2e^−16^). Net impregnation with permethrin however did not significantly affect mortality, with a mean mortality of 1.80% in females exposed to ethanol (95% CI [0.27–3.34]) and of 2.71% in females exposed to permethrin (95% CI [−0.13–5.55]; *X*² = 0.86, Df = 1, P = 0.35).

After the first exposure, blood-fed females were kept and exposed to a second blood-meal through a net treated with the same dose of permethrin, after 3 or 4 days. For this second blood-meal, a subset of the females was followed individually whereas the others were exposed to the blood-meal by groups of 25. For the second exposure, both groups of naive and permethrin-pre-exposed mosquitoes were divided into two subgroups for the second exposure: half was exposed to ethanol and the other half to permethrin. The following four treatments were then obtained regarding the treatment mosquitoes received at each exposure: (i) Ethanol - Ethanol (EE), (ii) Permethrin - Ethanol (PE), (iii) Ethanol - Permethrin (EP) and (iv) Permethrin - Permethrin (PP).

### Effect of a first exposure to permethrin on the blood-feeding success at the second exposure to a permethrin-treated net

**Figure 1:**
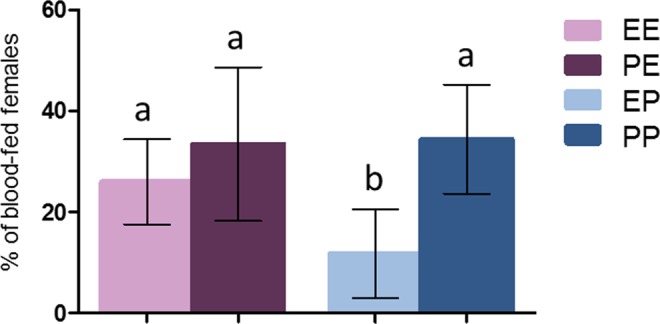
Proportion of blood fed *An. gambiae* during the second blood meal for each treatment. E = ethanol, P = Permethrin. The first letter show treatment received at the first exposure, the second letter show treatment received at the second exposure. Results are presented as mean ± 95% confidence intervals (95% CI). Different letters indicate significant differences (post hoc chi-squared tests with a Bonferroni correction, P < 0.05).

Blood-feeding success at the second blood-meal was tested for the 753 females that blood-fed at the first blood-meal. 436 were tested for the grouped exposure and 317 for the individual exposure, across 7 replicates. A successful blood-meal obtained upon a permethrin-treated net significantly affected behavior at the second exposure, with a significant interaction observed between the first and the second exposure (*X*² = 4.45, Df = 1, P = 0.035). When comparing the four treatments (EE, EP, PE, PP), females pre-exposed to permethrin displayed significantly higher blood-feeding rates in presence of permethrin at the second exposure (PP, mean = 34.38%, 95% CI [23.60–45.16]) compared to females pre-exposed to control nets (EP, mean = 11.80%, 95% CI [3.01–20.60]) (P < 0.001). Moreover, the proportion of females pre-exposed to permethrin that successfully blood-fed in presence of permethrin at the second exposure (PP) was not significantly different from the proportion of females that fed on control nets at the second exposure (EE, mean = 25.97%, 95% CI [17.51–34.43], P = 0.078 and PE, mean = 33.44%, 95% CI [18.25–48.62], P = 0.1). Also, a trend was observed for an increased blood-feeding rate upon a control net in permethrin pre-exposed females (PE) compared to control ones (EE), although difference was not significant (P = 0.058). Among females pre-exposed to control nets, exposure to permethrin at the second blood-meal (EP) significantly affected blood-feeding, with a mean 50.46% inhibition (95% CI [20.53–80.38]) compared to control group (EE) (P = 0.027) (Fig. 1).

#### Analysis on the offspring

**Figure 2:**
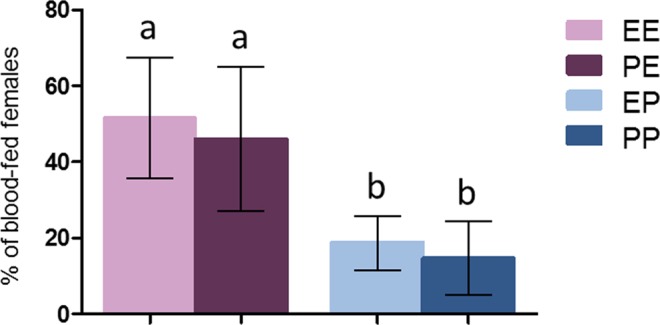
Assay on the offspring: proportion of blood fed *An. gambiae* depending on their mother’s treatment. E = ethanol, P = Permethrin. The first letter show treatment received by mothers, the second letter show treatment received by daughters. Results are presented as mean ± 95% confidence intervals (95% CI). Different letters indicate significant differences (post hoc chi-squared tests with a Bonferroni correction, P < 0.05).

To test the potential effect of a genetic selection for permethrin insensitivity that could have affected the observed female blood-feeding success at the second exposure, offspring from females exposed to permethrin and from naïve females were tested for their response to a blood-meal upon either a permethrin-treated net or a control net. Analysis on the offspring was performed on 1,027 females across 7 replicates. Results showed that blood-feeding rates of females whose mothers were exposed to permethrin did not differ neither on control nets (PE, mean = 45.95%, 95% CI [27.02–64.88], P = 0.43), nor on permethrin-treated nets (PP, mean = 14.64%, 95% CI [4.96–24.31], P = 0.40) compared to control females (EE, mean = 51.52%, 95% CI [35.62–67.41]; EP, mean = 18.74%, 95% CI [11.62–25.86]) (Fig. 2), suggesting that the effect of pre-exposure on feeding success at the second blood meal is not due to genetic selection.

#### Wing measurements

**Figure 3:**
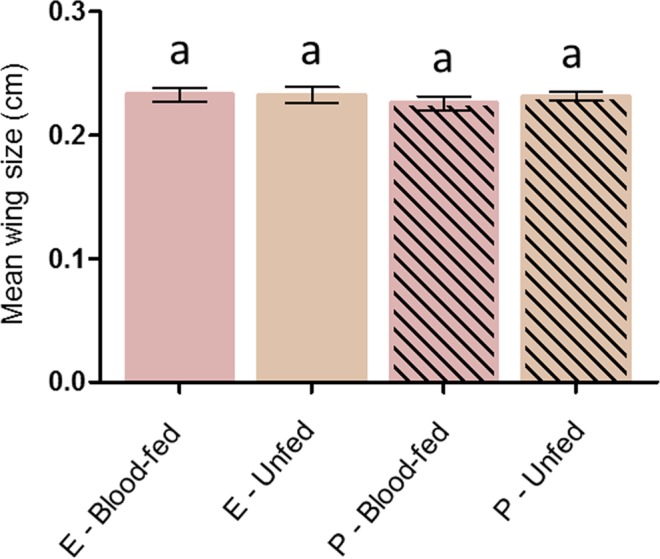
Wing size measurement of *An. gambiae* after exposure to ethanol or permethrin - treated nets. E = ethanol, P = Permethrin. Results are presented as mean ± 95% confidence intervals (95% CI). Different letters indicate significant differences (post hoc chi-squared tests with a Bonferroni correction, P < 0.05).

To test the potential bias of a physiological selection for permethrin insensitivity^36^, size of surviving females was compared regarding the exposure they received (*i.e*. ethanol or permethrin) and their blood-feeding status. Wing measurements were performed in a subset of 335 females across 3 replicates. Data showed no significant differences in female body size regarding the treatment they received (*X*² = 1.30, Df = 1, P = 0.25). Yet, females that successfully blood-fed upon a permethrin treated net did not significantly differ in their body size neither from females that unsuccessfully blood-fed (P-blood - fed, mean = 0.23, 95% CI [0.22–0.23]; P-unfed, mean = 0.23, 95% CI [0.23–0.24]; P = 0.1), nor from females that were exposed to ethanol-treated nets and successfully or unsuccessfully blood-fed (E- blood - fed, mean = 0.23, 95% CI [0.23–0.24]; P = 0.81; E-unfed, mean = 0.23, 95% CI [0.23–0.24]; P = 0.71) (Fig. 3).

### Effect of permethrin on life-history traits after the first blood-meal

To assess the residual impact of permethrin exposure on mosquitoes, female life-history traits related to fecundity, fertility and survival were measured after the first exposure: size of the blood-meal (using quantity of excreted hematin as a proxy), number of eggs laid (*i.e*. fecundity), number of descendants produced (*i.e*. fertility), proportion of eggs that reach adult stage among the offspring (*i.e*. emergence rate) and proportion of surviving females 3 days post-exposure. Blood-meal size was assessed in a subset of 75 females followed individually across 2 replicates. Fecundity, fertility, emergence rate of the offspring and proportion of surviving females 3 days post-exposure were measured in all the 1,103 blood-fed females followed by group of 30 across 7 replicates. Fecundity, fertility and emergence rate were expressed as a mean per female.

**Figure 4:**
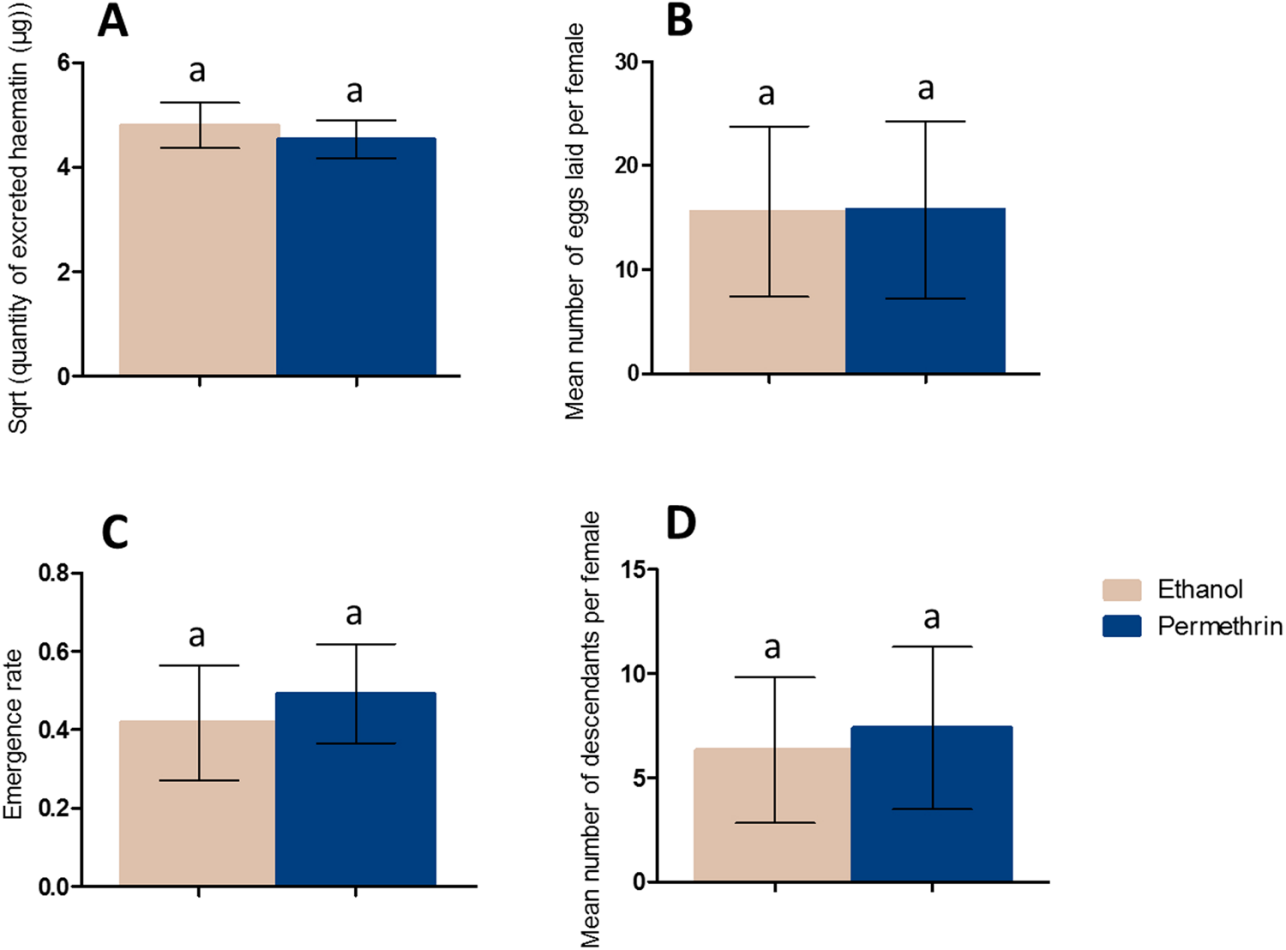
Effect of permethrin exposure on mosquito life history traits after the first blood-meal: volume of blood ingested (**A**), fecundity (**B**), offspring emergence rate (**C**) and fertility (**D**). Results are presented as mean ± 95% confidence intervals (95% CI). Different letters indicate significant differences (post hoc chi-squared tests with a Bonferroni correction, P < 0.05).

Life-history traits after the first exposure were not affected by permethrin treatment. Indeed, a first exposure to permethrin did not influence the size of the blood-meal (Control, mean = 4.80 μg, 95% CI [4.37–5.23]; Permethrin - treated, mean = 4.53 μg, 95% CI [4.16–4.89]; *X*² = 1.18, Df = 1, P = 0.28) (Fig. 4A), fecundity (Control, mean = 15.6 eggs per female, 95% CI [7.40–23.8]; Permethrin - treated, mean = 15.75 eggs per female, 95% CI [7.25–24.26]; *X*² = 0.002, Df = 1, P = 0.96) (Fig. 4B), emergence rate of the offspring (Control, mean = 0.42 adult per laid egg, 95% CI [0.27–0.56]; Permethrin - treated, mean = 0.49 adult per laid egg, 95% CI [0.37–0.62]; *X*² = 1.33, Df = 1, P = 0.25) (Fig. 4C), nor fertility (Control, mean = 6.34 descendants per female, 95% CI [2.85–9.83]; Permethrin – treated, mean = 7.39 descendants per female, 95% CI [3.50–11.29]; *X*² = 0.33, Df = 1, P = 0.57) (Fig. 4D). Survival 3 days post-exposure was neither affected by permethrin treatment (Control, mean = 89% of surviving females 3 days after exposure, 95% CI [82.21–95.79]; Permethrin - treated, mean = 87.90% of surviving females 3 days after exposure, 95% CI [79.64–96.17]; *X*² = 0.012, Df = 1, P = 0.91).

### Effect of permethrin on life-history traits after the second blood-meal

To assess the impact of two exposures to permethrin on mosquitoes, life-history traits were also recorded during and after the second exposure. Mosquito flight activity during the blood-meal and exposure was recorded in individual-exposed mosquitoes, using infrared beams arrays (see Supplementary Figs S1 and S2). Blood-meal size, fecundity, fertility, oviposition rate (*i.e*. proportion of females that oviposited), female survival and offspring emergence rate were measured in females followed individually after exposure, across 7 replicates. Mosquito activity was assessed in a subset of 91 individualized females during the second blood-feeding. Size of the blood-meal, oviposition rate and survival were measured in 222 individualized females. Fecundity was estimated in the 118 females that oviposited. For fertility and emergence rate measurement, larvae were grouped by treatment so data is expressed as a mean per female.

#### Effect of the first exposure to permethrin on life-history traits following the second blood-meal

**Figure 5:**
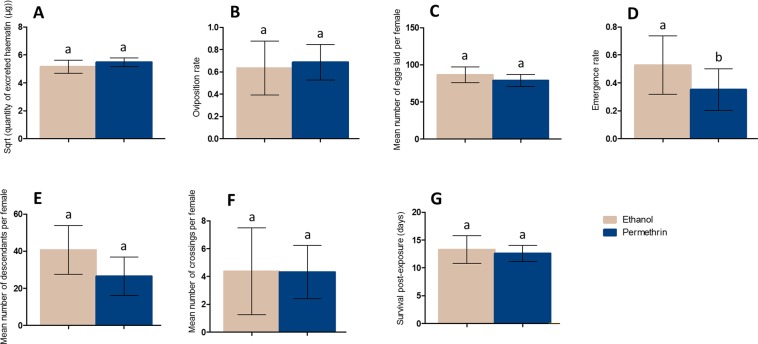
Effect of the first exposure to permethrin on life history traits ensuing the second blood-meal: volume of blood ingested (**A**), oviposition rate (**B**), fecundity (**C**), offspring emergence rate (**D**), fertility (**E**), activity (**F**) and survival post-exposure (**G**). For each trait, females were pooled considering the treatment they received at the first exposure. Results are presented as mean ± 95% confidence intervals (95% CI). Different letters indicate significant differences (post hoc chi-squared tests with a Bonferroni correction, P < 0.05).

A prior exposure to permethrin did not significantly affect blood-meal size (Control, mean = 5.15 μg, 95% CI [4.68–5.63]; Permethrin - treated, mean = 5.48 μg, 95% CI [5.17–5.79]; *X*² = 0.0018, Df = 1, P = 0.97) (Fig. 5A), oviposition rate (Control, mean = 0.63 ovipositing female per blood-fed female, 95% CI [0.39–0.88]; Permethrin - treated, mean = 0.69 ovipositing female per blood-fed female, 95% CI [0.53–0.85]; *X*² = 2.28, Df = 1, P = 0.13) (Fig. 5B) nor fecundity (Control, mean = 86.76 eggs per female, 95% CI [76.04–97.47]; Permethrin - treated, mean = 78.95 eggs per female; 95% CI [70.89–87.02]; *X*² = 0.27, Df = 1, P = 0.60) (Fig. 5C). However, for a similar number of eggs laid, the proportion of eggs that reach adult stage was reduced by a 34% in females pre-exposed to permethrin (Control, mean = 0.53 adult per laid egg, 95% CI [0.32–0.74]; Permethrin - treated, mean = 0.35 adult per laid egg, 95% CI [0.20–0.50]; *X*² = 5.34, Df = 1, P = 0.021), irrespectively of the treatment they received at the second exposure (Fig. 5D). Although a trend was observed for a reduced number of descendants in females pre-exposed to permethrin, this was not significant (Control, mean = 40.73 descendants per female, 95% CI [27.61–53.86]; Permethrin - treated, mean = 26.55 descendants per female, 95% CI [16.17–36.93]; *X*² = 3.63, Df = 1, P = 0.057) (Fig. 5E). Besides, a prior exposure to permethrin did not affect mosquito flight activity during subsequent blood-feeding (Control, mean = 4.37 crossings per female, 95% CI [1.26–7.48]; Permethrin - treated, mean = 4.32 crossings per female, 95% CI [2.42–6.22]; *X*² = 0.015, Df = 1, P = 0.90) (Fig. 5F), nor survival (Control, mean = 13.29 days, 95% CI [10.81–15.77]; Permethrin - treated, mean = 12.61 days, 95% CI [11.17–14.05]; *X*² = 0.42, Df = 1, P = 0.52) (Fig. 5G).

#### Effect of the second exposure to permethrin on life-history traits following the second blood-meal

**Figure 6:**
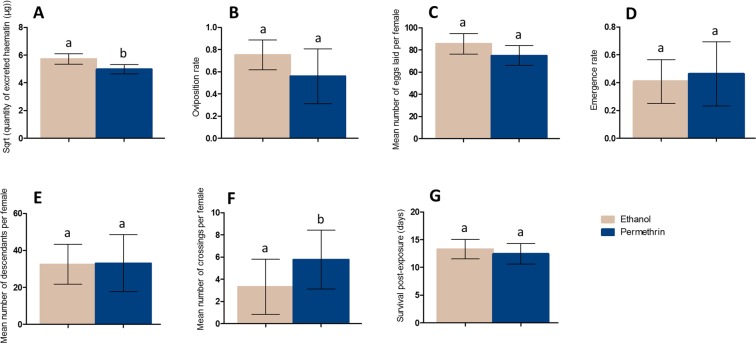
Effect of the second exposure to permethrin on life history traits ensuing the second blood-meal: volume of blood ingested (**A**), oviposition rate (**B**), fecundity (**C**), offspring emergence rate (**D**), fertility (**E**), activity (**F**) and survival post-exposure (**G**). For each trait, females were pooled considering the treatment they received at the second exposure. Results are presented as mean ± 95% confidence intervals (95% CI). Different letters indicate significant differences (post hoc chi-squared tests with a Bonferroni correction, P < 0.05).

Exposure to permethrin at the second blood-meal significantly reduced blood-meal size by a 13% (Control, mean = 5.72 μg, 95% CI [5.34–6.11]; Permethrin - treated, mean = 4.98 μg, 95% CI [4.66–5.31]; *X*² = 18.0021, Df = 1, P = 2.21e^−5^), irrespectively of the first exposure (Fig. 6A). The second exposure to permethrin did not affect subsequent oviposition rate (Control, mean = 0.75 ovipositing female per blood-fed female, 95% CI [0.61–0.88]; Permethrin - treated, mean = 0.55 ovipositing female per blood-fed female, 95% CI [0.31–0.80]; *X*² = 2.98, Df = 1, P = 0.084) (Fig. 6B), fecundity (Control, mean = 85.50 eggs per female, 95% CI [76.29–94.71]; Permethrin – treated, mean = 74.94 eggs per female, 95% CI [66.07–83.81]; *X*² = 1.82, Df = 1, P = 0.18) (Fig. 6C), proportion of eggs that reach adult stage (Control, mean = 0.41 adult per laid egg, 95% CI [0.25–0.57]; Permethrin - treated, mean = 0.46 adult per laid egg, 95% CI [0.23–0.69]; *X*² = 3.33, Df = 1, P = 0.068) (Fig. 6D), nor fertility (Control, mean = 32.51 descendants per female, 95% CI [21.79–43.24]; Permethrin – treated, mean = 33.03 descendants per female, 95% CI [17.60–48.45]; *X*² = 0.14, Df = 1, P = 0.71) (Fig. 6E). However, mosquito activity during the second blood feeding was significantly influenced by permethrin treatment, with an increased activity of 73% in females exposed to permethrin (Control, mean = 3.33 crossings per female, 95% CI [0.84–5.8]; Permethrin – treated, mean = 5.78 crossings per female, 95% CI [3.12–8.43]; *X*² = 4.22, Df = 1, P = 0.04) (Fig. 6F). Exposure to permethrin at the second blood-meal did not affect mosquito survival (Control, mean = 13.29 days, 95% CI [11.53–15.05]; Permethrin – treated, mean = 12.43 days, 95% CI [10.57–14.29]; *X*² = 0.044, Df = 1, P = 0.83) (Fig. 6G).

## Discussion

In this work, we assessed the effects of two exposures to permethrin on its efficacy to inhibit a blood-meal in the malaria mosquito *An. gambiae* carrying pyrethroid resistance alleles. We also measured the impact of permethrin exposure on life-history traits related to fecundity, fertility and survival.

Our results evidenced decreases in permethrin irritant effect after a prior exposure in the malaria mosquito *An. gambiae* carrying homozygote *kdr* pyrethroid resistance alleles. After having successfully obtained a blood-meal upon a permethrin-treated net despite irritancy, resistant females were not affected anymore by permethrin net treatment in their success to feed at the subsequent exposure. Wing measurements and assays on the offspring showed no association with a genetic or physiological selection for permethrin-insensitive females. Also, in this study, permethrin pre-exposed females showed no differences in their response to control untreated net than females pre-exposed to control net. This suggests that the observed effects after initial permethrin exposure are not due to deleterious effects on mosquito fitness that would increase the need for blood-feeding, but rather to a reduction of permethrin effect on mosquito behavior. Possible explanations for these behavioral modifications could, first, lie in the effects of hyper excitation and irritancy that characterize pyrethroids mode of action in insects^27,37,38^. Yet, the contact with the insecticide at the first exposure could have induced alterations in sensory receptors or in the neuronal activity at the central level, resulting in an absence of detection at the subsequent exposure. Consistently, permethrin has been shown to disrupt chemical communication and cognition in other insect species^39–41^. In mosquitoes, behavioral responses to host-associated attractants were reduced 24 hours after exposition to permethrin^42^. Also, modification of attraction to experimental oviposition sites after exposure to volatile pyrethroid, transfluthrin, have been documented in *Ae. aegypti*^43^. Pyrethroid and particularly permethrin exposure may then induce long-term alteration in sensory system. As no host attractants were used in this study, we hypothesize that pre-exposure to permethrin could have altered its detection at the subsequent exposure. It is worth noting that the behavior observed could also be the result of sensory habituation, *i.e*. when sensory receptors stop responding to a stimulus after being continuously stimulated. Although the cases of habituation documented in insects reported that behavior was impacted for a few minutes after exposure^44–46^, this hypothesis deserves to be considered. Further studies are then needed to depict the effects of multiple exposures to permethrin on pyrethroid-resistant mosquitoes, and on their subsequent ability to detect pyrethroids, hosts odors, mates, and to find food or shelters. Secondly, the observed behavioral modifications could be due to an enhanced detoxifying activity after the first exposure, resulting in a lower sensibility at the second exposure. Yet, exposure to a chemical has been shown to induce the production of detoxifying enzymes in insects^47^, sometimes conferring an increased protection at the subsequent exposure^48^, a phenomenon called “enzyme induction”. This hypothesis however needs further investigation, as the role of enzyme induction has never been investigated in this context. Finally, a prior exposure to permethrin could have triggered learning mechanisms in female mosquitoes. Indeed, mosquitoes are known to have the ability to memorize the association between a stimulus and a reward when these are concomitant^49–51^. They could then have associated the presence of permethrin with the probability of obtaining a blood-meal, overstepping the deterrent effects of the insecticide during the second exposure. Consistently, learning abilities have been evidenced in *An. gambiae*, in which the associative learning was retained for up to three days^50^. This hypothesis however raises some questions. Yet, is it not known whether a single exposure is sufficient to trigger learning, nor if mosquitoes can learn from an irritant compound such as permethrin. Also, to our knowledge, there is no documentation about the pathways involved in permethrin detection in mosquitoes. This chemical might be detected through the gustatory pathway due to its irritant effects^38^, but the gustatory neurons involved have not been identified. In the same way, although the involvement of the olfactory pathway is suspected^52,53^, evidences for an olfactory detection of permethrin are still lacking. In this context, if learning took place, it is of crucial interest to investigate which pathway is involved, and the effects of pyrethroid resistance on learning. These data would, in the long-term, help to understand the impact of ITNs use on dynamics of pathogens transmission.

Our results show that, under our experimental design, life-history traits recorded were not affected by multiple exposures to permethrin. Indeed, quantity of ingested hemoglobin, oviposition rate, fecundity, fertility, survival and flight activity were not modified after prior exposure to the insecticide. Also, during the second blood-meal, females exposed to permethrin displayed increased activity associated with reduced quantity of ingested hemoglobin. This confirms that permethrin is irritant even against pyrethroid-resistant mosquitoes^54^, which renders most difficult the blood-feeding and force them to make more attempts to fully engorge. However, this irritant effect did not affect subsequent fecundity nor fertility, as the total number of descendants produced was not significantly different between permethrin-exposed and control females. Consequently, in these experimental conditions, pyrethroid exposure did not impact the fitness of a colony carrying pyrethroid-resistant alleles. Furthermore, our results did not evidence any effect of the two exposures to permethrin on mosquito survival. This contrasts with the delayed mortality previously observed in insecticide-resistant *An. gambiae* after multiple exposures to pyrethroids^22^. These discrepancies may originate both in the use of different mosquito strains and insecticides tested between the studies. For instance, we used permethrin, which is known to be more irritant for mosquitoes than deltamethrin^13^. Altogether, our data show, with a pyrethroid-resistant strain of *An. gambiae*, both loss of permethrin efficacy after a prior exposure, and no residual effects of permethrin on mosquito life-history traits related to fecundity, fertility and survival. These data may have substantial consequences for vector control, as it suggests that the residual efficacy of permethrin-treated nets against mosquitoes carrying resistance alleles may be much lower than expected. ITNs may then not be efficient nor to inhibit blood-feeding, nor to kill pyrethroid resistant *Anopheles* and may consequently not protect people properly against infectious bites. This is particularly concerning in malaria endemic areas, where most of mosquitoes are resistant to pyrethroids^2^ and where ITNs are still main components of vector control. In this context, it is of crucial interest to understand the fundamental causes of these behavioral modifications, and if these changes could be observable under natural conditions. Additional experiments are then needed to depict the long-term effects of permethrin on pyrethroid-resistant mosquitoes with regard to nervous system, olfaction, gustation, and learning abilities. Also, investigating the combined effects of experience and infection would allow to model the consequences of the observed effects on pathogens transmission. These data provide great evidences for phenotypic plasticity in the malaria mosquito *An. gambiae*. They also highlight the need for rethinking the way we fight mosquito-borne diseases, and for developing new vector control tools that could complete or replace insecticides and help to reduce and interrupt pathogen transmission. An integrated, multifaceted approach is needed, with alternating tools that limit selective pressure then the spread of resistances.

## Methods

### Mosquito colony

Experiments were performed on *An. gambiae* females that had the opportunity to mate and have not been previously blood-fed. The used colony, named KdrKis strain, harbored the L1014F homozygote mutation (*kdr*-west allele) in the gene coding for the voltage-gated sodium channel, which confers resistance to pyrethroids and DDT. The colony was obtained by introgression into the susceptible genetic background of the Kisumu colony the *kdr*-west allele obtained from pyrethroid resistant mosquitoes in Kou Valley, Burkina Faso^55^. Insecticide resistance was confirmed by using insecticide bioassays and genetic verifications^56^. The colony does not carry any metabolic resistance. Mosquitoes were reared and tested in the technical/research platform dedicated on vectors at the Institut de Recherche pour le Développement (IRD) Centre, Montpellier, France. They were maintained at 27 °C and 80% of relative humidity with a L:D photoperiod of 14:10. Larvae were provided TetraMin (Tetra, Germany) and adults a 10% honey solution.

### First exposure to permethrin: blood-feeding through impregnated net

Permethrin efficacy was assessed regarding mosquito ability to blood-feed through an impregnated net. Experiments were performed by using the protocol previously described^57^. Batches of 25 females were distributed in paper cups (H: 10 cm, ⌀: 7 cm). Nets impregnated with permethrin emulsifiable concentrate (Peripel®) diluted in ethanol at the WHO recommended dose of 500 mg/m² for conventional net impregnation^12^ were then placed on the cups containing mosquitoes, covering the mesh that closes the cup. Control mosquitoes were exposed to nets treated with the solvent ethanol. Cups were put under glass feeders filled with rabbit blood and sealed on one end with parafilm membrane, so mosquitoes would contact the impregnated net while simultaneously blood-feeding. Mosquitoes were allowed to feed upon the treated/control net for one hour, after which the proportion of blood-fed females was recorded for each treatment. Blood-fed females were then placed in clean cups maintained in the same conditions as during the rearing for subsequent recording of life history traits. They were supplemented with 10% honey and were given the opportunity to oviposit.

### Second exposure to permethrin: blood-feeding through impregnated net

A second blood-meal through permethrin-treated net was provided 3 or 4 days after the first exposure. Each group of mosquitoes (control and permethrin-pre-exposed) was split into two subgroups for this second exposure: half was exposed to ethanol and the other half to permethrin. The following four treatments were then obtained regarding the treatment mosquitoes received at each exposure: (i) Ethanol - Ethanol (EE), (ii) Permethrin - Ethanol (PE), (iii) Ethanol - Permethrin (EP) and (iv) Permethrin - Permethrin (PP). During this blood-feeding, a subset of permethrin-pre-exposed and control mosquitoes were followed individually for each replicate in order to monitor their flight activity. All females (individual and grouped) were provided the second blood-meal following the same protocol as described above. After one hour, the number of blood-fed females was counted, and they were all kept singly in the same conditions as during the rearing in order to monitor life-history traits.

### Life-history traits recording

Several life-history traits were recorded and used as fitness indicators to assess the long-term impact of permethrin exposure on mosquitoes, according to procedures previously described^57^: blood-meal size (using excreted hematin as a proxy^58^), fecundity, fertility and emergence rate of the offspring were recorded both after the first and the second exposure. Survival 3 days post-exposure was assessed after the first exposure; oviposition rate and survival were assessed after the second exposure. Besides, as the irritancy of permethrin may affect the success to blood-feed and then possibly increase the energy spent to take a blood-meal, female flight activity during the second blood-feeding and exposure was measured in individually-tested mosquitoes. To do this, we used a locomotor activity monitor system (TriKinetics, Waltham, MA, USA). The device consists of a series of infrared LEDs placed around a 30 ml tube where the mosquito is placed, and each time it comes back and forth to the provided blood, interruption of the beams was recorded. The number of interruptions (*i.e*. crossings) was used as a proxy of the mosquito flight activity during the assay (see Supplementary Figs S1 and S2). Also, the selection of blood-fed females after the first exposure could genetically or physiologically select for permethrin insensitivity and then affect the feeding success under permethrin presence at the second exposure. To control for this potential bias, assays on the offspring and female size measurement were performed.

#### Assays on the offspring

Offspring from all the tested females was kept and the descendant females were exposed to a blood-meal in presence - absence of permethrin according to the protocol used for the second blood-meal of their mothers. Comparisons of the blood-feeding success through permethrin-impregnated nets between offspring females from permethrin-exposed mothers and from control mothers were thus carried out.

#### Wing size

Female size is thought to affect its susceptibility to chemicals^36^. The selection of blood-fed females after the first exposure to permethrin could lead to a selection of the largest females and then the most resistant to permethrin, affecting blood-feeding success at the second exposure. To control for this potential confounding factor, size measurements were performed on females exposed to either permethrin or ethanol, using wing size as an indicator of female size. After blood-feeding through impregnated net, all females (control and exposed to permethrin, fed and unfed) were kept and frozen at −30 °C. One wing of each female mosquito was then cut and scanned with a high performance scanner (ImageScanner III, GE Healthcare, Buc, France) at a resolution of 6,000 dpi. Wing size was assessed by measuring the distance from the axillary incision to the tip of the wing, excluding the wing fringe^59^. Wing size was determined by using the open source ImageJ software^60^, after which a comparison was established regarding treatment and blood-feeding status.

### Statistical analysis

All statistical analyses were performed using the software R 3.3.2^61^.

#### Blood-feeding

The proportion of blood-fed females after the first and the second blood-meal was compared between treatments using generalized linear mixed-effects model (glmer, binomial distribution, logit link, lme4 package^62^). For these analyses, replicate was coded as a random factor, as replicates were conducted on different days. Post-hoc comparisons between the four groups of treatment at the second exposure were performed using multiple comparisons (Tukey’s tests, multcomp package^63^). The effect of mother exposure on the success of blood-feeding of the offspring was also assessed by glmer with binomial distribution, coding replicate as a random factor.

#### Life-history traits

In a first analysis, we tested for the effect of the first exposure on subsequent life history traits. Mortality during exposure and mortality 3 days post-exposure were analyzed using glmer with binomial distribution, with replicate coded as a random factor. The quantity of excreted haematin was assessed using linear mixed-effects model with a Gaussian distribution, after sqrt transformation and confirmation of data normality (lmer, lme4 package). Fecundity, fertility and wing size were also analyzed using lmer with a Gaussian distribution, without data transformation. For all these models, replicate was coded as a random factor. Emergence rate (proportion of eggs that reached adult stage) was analyzed using glm with quasibinomial error distribution to account for overdispersion, with replicate coded as a fixed factor.

In a second analysis, we tested for the effect of the first and the second exposure on each life history trait considered subsequent to the second exposure. Quantity of excreted haematin, emergence rate and fertility were assessed using the same methodology as described above. Activity during the second blood-meal and fecundity were estimated by using generalized linear mixed models using AD Model Builder to account for overdispersion (glmmadmb, negative binomial distribution, glmmADMB package^64^). Oviposition rate (the proportion of females that oviposited) was assessed by using glmer with binomial distribution. Survival was evaluated with a mixed effects Cox proportional hazards regression model (packages survival, coxme^65,66^). For all these models, replicate was coded as a random factor. Several explanatory variables were included in these models to control for their influence: mosquito age, the day after the first exposure (3 or 4), and the type of exposure (grouped or individual). The contribution of each explanatory variable was assessed sequentially using anova function, with non-significant terms removed from the model. Model selection was performed using AIC and analysis of the residuals (plotresid, RVAideMemoire package^67^). Results are presented as mean ± 95% confidence intervals (95% CI).

## Supplementary information

Supplementary figure S1 and Supplementary Figure S2

## Data Availability

The datasets used and/or analyzed during the current study are available from the corresponding author on reasonable request.
