# Supplementary material for: Prior contact with permethrin decreases its irritancy at the following exposure among a pyrethroid-resistant malaria vector Anopheles gambiae
Source: Sci Rep. 2019 Jun 3;9:8177. doi: 10.1038/s41598-019-44633-1 (PMC6546682; doi:10.1038/s41598-019-44633-1)

Supplementary information

Prior contact with permethrin decreases its irritancy at the following exposure among a pyrethroid-resistant malaria vector *Anopheles gambiae*

Margaux Mulatier[1,2*], Cédric Pennetier[1,3], Angélique Porciani[1], Fabrice Chandre[1], Laurent Dormont[2], Anna Cohuet[3]

[1]: MIVEGEC, IRD, CNRS, Univ. Montpellier, Montpellier, France.

[2]: CEFE, Univ Paul Valéry Montpellier 3, CNRS, Univ Montpellier, EPHE, IRD, Montpellier, France

[3]: Institut Pierre Richet, Bouaké, Côte d'Ivoire

* Corresponding author: margaux.mulatier@ird.fr

Supplementary figure S1: Representation of the Locomotor Activity Monitor. Mosquitoes are individually placed in 30 mL tubes surrounded by a series of infrared LEDs. These LEDs count the number of vertical crossings a mosquito make to come back and forth to the provided blood.

Supplementary figure S2: Representation of the Locomotor Activity Monitor. Mosquitoes are individually placed in 30 mL tubes surrounded by a series of infrared LEDs. These LEDs count the number of vertical crossings a mosquito make to come back and forth to the provided blood.

Supplementary Figure S1

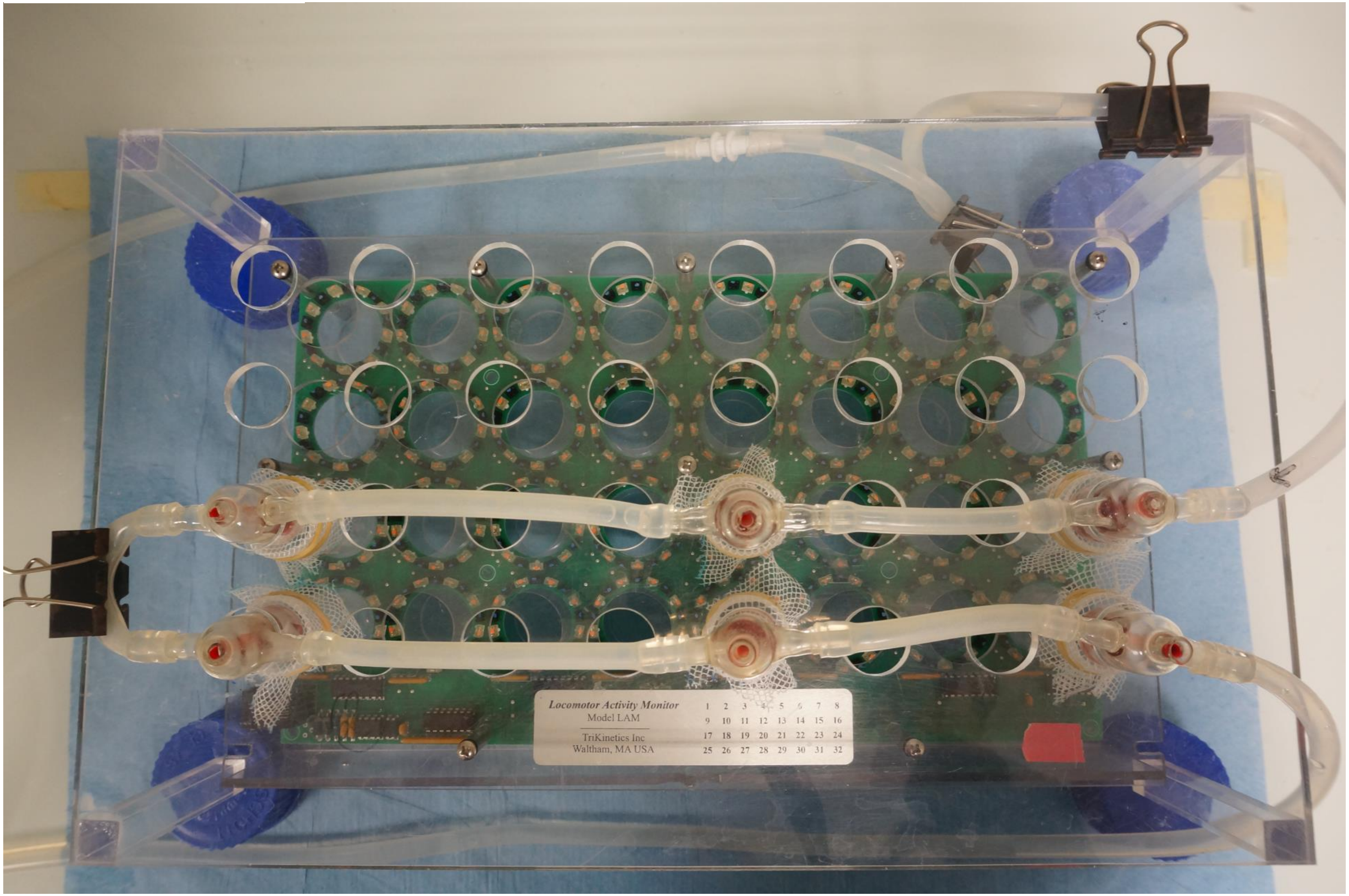

Supplementary Figure S2

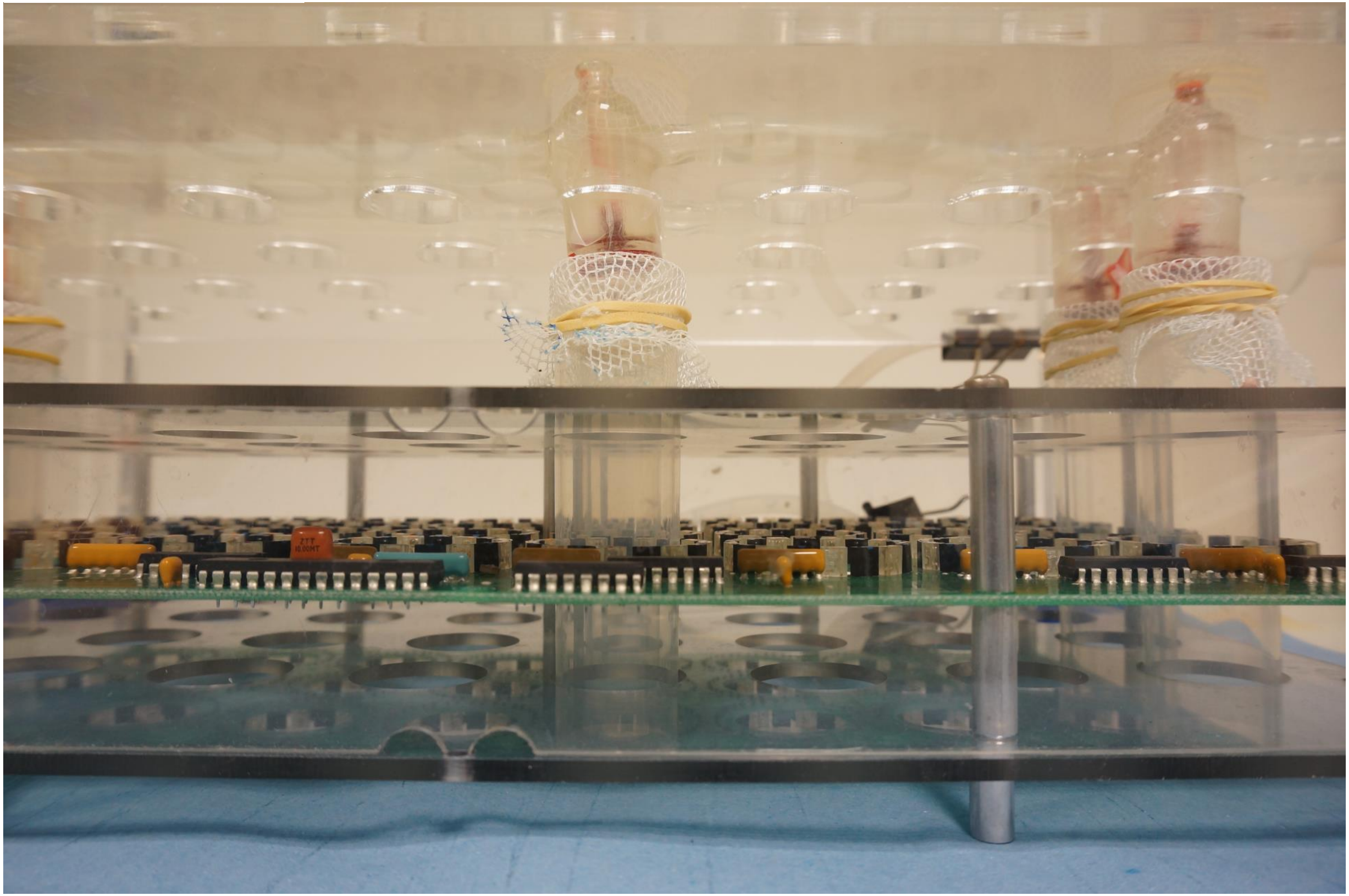

Supplement: Supplementary file 1 — Supplementary figure S1 and Supplementary Figure S2 [file 41598_2019_44633_MOESM1_ESM.pdf]
